# *Notes from the Field:* Increase in Pediatric Invasive Group A *Streptococcus* Infections — Colorado and Minnesota, October–December 2022

**DOI:** 10.15585/mmwr.mm7210a4

**Published:** 2023-03-10

**Authors:** Meghan Barnes, Erin Youngkin, Jennifer Zipprich, Kayla Bilski, Christopher J. Gregory, Samuel R. Dominguez, Erica Mumm, Melissa McMahon, Kathryn Como-Sabetti, Ruth Lynfield, Sopio Chochua, Jennifer Onukwube, Melissa Arvay, Rachel Herlihy

**Affiliations:** ^1^Colorado Department of Public Health and Environment; ^2^Minnesota Department of Health; ^3^National Center for Immunization and Respiratory Diseases, CDC; ^4^University of Colorado School of Medicine and Children’s Hospital Colorado, Aurora, Colorado.

During fall 2022, a resurgence of invasive group A *Streptococcus* (iGAS) infection in children and adolescents was observed in two of CDC’s Emerging Infections Program (EIP)* surveillance sites: Colorado (Denver metropolitan area) and Minnesota (entire state). This increase followed historic declines in invasive bacterial diseases during 2020, concurrent with mitigation strategies implemented during the COVID-19 pandemic^†^ (*1*). Whereas reports of iGAS increased among all age groups, including adults, the increase among children and adolescents was notable, occurred earlier than seasonal increases during previous years, and accompanied a resurgence in hospitalizations for respiratory viral illnesses such as respiratory syncytial virus (RSV) and influenza. Viral infections, such as influenza and varicella, have been identified as risk factors for iGAS infection in children, adolescents, and adults (*2*) and can be reduced by vaccination.

Surveillance for iGAS is conducted by 10 U.S. sites as part of EIP’s Active Bacterial Core surveillance (ABCs).^§^ An analysis of cases among Colorado and Minnesota EIP site residents aged <18 years who met criteria for iGAS^¶^ was conducted using ABCs data from the Colorado and Minnesota surveillance sites. Case counts, age distribution, and clinical characteristics of patients with iGAS infection were compared over three periods: baseline (January 1, 2016–December 31, 2019), pandemic (January 1, 2020–December 31, 2021), and recent increase (October 1–December 31, 2022). This activity was reviewed by CDC and was conducted consistent with applicable federal law and CDC policy.**

During October 1–December 31, 2022, a combined total of 34 cases was reported in the Colorado and Minnesota ABCs sites. In comparison, a 3-month average of 11 cases and four cases were observed during the same period in 2016–2019 and 2020–2021, respectively. Colorado patients identified during the recent increase were younger (median age = 3.1 years) than were those during the baseline period (5.6 years) and the pandemic period (6.2 years); this was not observed in Minnesota (median age = 4.0, 6.0, and 6.5 years in the baseline, pandemic, and recent increase periods, respectively). Two deaths (one each in Colorado and Minnesota) were noted during the recent increase period; overall, during 2016–2021, five deaths occurred (one in Colorado and four in Minnesota). Frequency of intensive care unit admission and length of hospital stay were similar during the recent increase (35.3% [12 of 34 patients]; 4.5 days) and baseline periods (34.4% [62 of 180], 5.0 days).^††^ Most cases (73.5% [25 of 34]) that occurred during the recent increase were in children and adolescents without underlying medical conditions.

Among the 34 cases that occurred during the recent increase, 21 (61.8%) patients had an upper respiratory tract infection noted within the 2 weeks preceding their iGAS infection, six (17.6%) reported sore throat, and seven (20.6%) reported no preceding illness. Fifteen (44.1%) patients received positive test results for one or more respiratory viral pathogen during the 2 weeks before, or concurrent with, their iGAS infection. Viral respiratory pathogens identified included RSV (six, 17.6%), influenza A or B (six, 17.6%), and SARS-CoV-2 (three, 8.8%).^§§^ Comparison of pediatric iGAS case counts, and influenza and RSV hospitalization rates during 2016–2022 showed an increase in iGAS infections coinciding with seasonal peaks in RSV and influenza hospitalization rates during most years except in 2021, when influenza and RSV hospitalizations were lower than those in previous or subsequent years (Figure).

**FIGURE:**
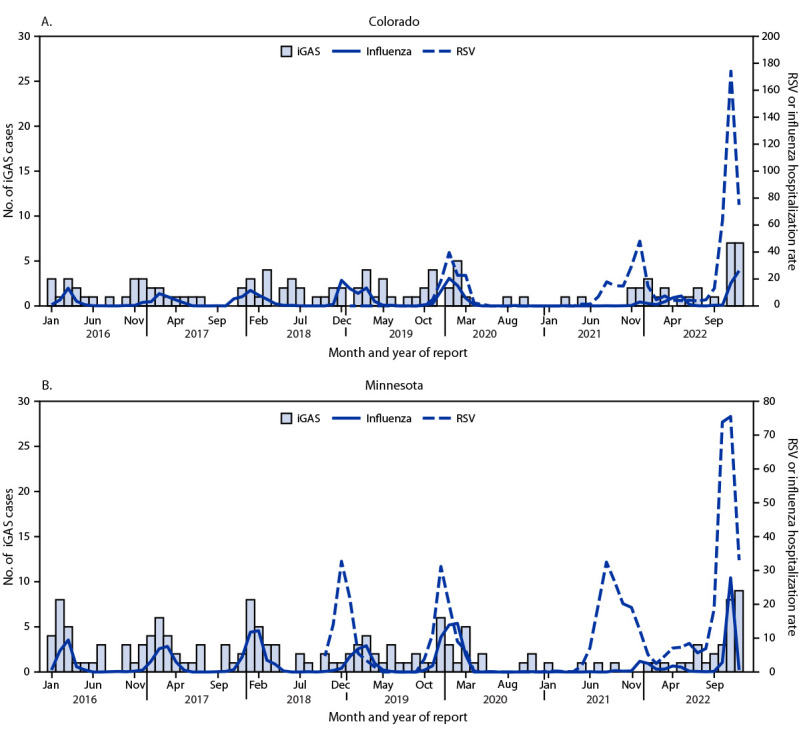
Cases of invasive group A *Streptococcus* infections* and hospitalization rates^†^ for influenza^§^ and respiratory syncytial virus^¶^ among children and adolescents aged <18 years — Colorado and Minnesota, January 2016–December 2022** **Abbreviations**: iGAS = invasive group A *Streptococcus* infection; RSV = respiratory syncytial virus. * iGAS infections were identified through each state’s Emerging Infections Program Active Bacterial Core surveillance systems. Cases in Colorado are from the Denver metropolitan area; cases in Minnesota throughout the state are reportable to the Minnesota Department of Health. ^†^ Hospitalizations per 100,000 population. ^§^ Colorado influenza hospitalizations are reported from the Denver metropolitan area, and rates in children and adolescents aged <18 years were calculated using age-specific and geographically defined population data obtained from the Colorado Department of Local Affairs, Demography Office. Influenza hospitalizations in Minnesota throughout the state are reportable to the Minnesota Department of Health; Minnesota influenza hospitalization rates in children and adolescents aged <18 years were calculated using age-specific and statewide population data obtained from CDC WONDER. ^¶^ RSV hospitalizations in Colorado were from the Denver metropolitan area; RSV hospitalization rates in children and adolescents aged <18 years were calculated using age-specific and Denver metropolitan population data obtained from the Colorado Department of Local Affairs, Demography Office. Colorado RSV hospitalization data are available during July 2019–December 2022. Minnesota RSV hospitalization rates are from the seven-county Twin Cities metropolitan area; rates in children and adolescents aged <18 years were calculated using age-specific and seven-county metropolitan population data obtained from CDC WONDER. Minnesota RSV hospitalization data were available during October 2018–December 2022. ** COVID-19 cases were not included because of the short period for which data were available and the variations in testing practices and surveillance catchment areas that limit the comparability of data.

Among the 26 (76%) iGAS cases from the recent increase period with M protein gene^¶¶^ (*emm*) typing results available, 22 (85.0%) were type 1 (nine, 34.6%) or type 12 (13, 50.0%); these were also the two most common types detected during the baseline period (55.1% type 1; 17.9% type 12). Whole genome sequencing results did not indicate changes in predicted antibiotic susceptibility (*3*) compared with earlier years or expansion of a single clone. Twenty-three isolates were predicted to be susceptible to all antimicrobials; one type 12 isolate was resistant to erythromycin, and two type 77 isolates were resistant to erythromycin, clindamycin, and tetracycline.

The increase in pediatric iGAS cases reported during fall 2022 is important for understanding the impact of the COVID-19 pandemic on the epidemiology of iGAS (*1*). Increased activity of respiratory viruses, in combination with reduced exposure to GAS and associated development of protective immunity to common *emm* types during the COVID-19 pandemic (*4*), might have predisposed children to iGAS infection when pandemic restrictions were lifted. The proportion of patients with preceding or concurrent influenza infections suggests that influenza vaccination might reduce the risk for iGAS, as has been demonstrated for varicella vaccination (*5*). Clinicians should consider iGAS as a possible cause of severe illness in children, adolescents, and adults, particularly among patients at increased risk,*** and offer influenza and varicella vaccination to eligible persons who are not up to date.

## Footnotes

* The Emerging Infections Program is a network of 10 state health departments (program sites) funded by CDC’s Division of Preparedness and Emerging Infections that collaborates with academic institutions and other public health stakeholders to address emerging infections. https://www.cdc.gov/ncezid/dpei/eip/index.html

† https://www.cdc.gov/abcs/reports-findings/data-2020.html (Accessed February 7, 2023).

§ https://www.cdc.gov/abcs/methodology/case-def-ascertain.html (Accessed January 30, 2023).

¶ Group A *Streptococcus* isolated or pathogen-specific nucleic acid detected using a validated molecular test in a specimen obtained from a normally sterile body site, or group A *Streptococcus* isolated from a wound culture and accompanied by necrotizing fasciitis or streptococcal toxic shock syndrome.

** 45 C.F.R. part 46.102(l)(2), 21 C.F.R. part 56; 42 U.S.C. Sect. 241(d); 5 U.S.C. Sect. 552a; 44 U.S.C. Sect. 3501 et seq.

†† A comparison with pandemic numbers is not provided because of the small number of cases during 2020–2021.

§§ Other respiratory pathogens detected included parainfluenza (two) and coronavirus other than SARS-CoV-2 (one).

¶¶ The M protein gene (*emm*) encodes the cell surface M virulence protein and forms the basis for the most widely used iGAS strain subtyping method.

*** Persons at increased risk of iGAS include those aged ≥65 years; American Indian or Alaska Native persons; residents of long-term care facilities; those with medical conditions such as diabetes, malignancy, immunosuppression, chronic kidney, cardiac, or respiratory disease; those with wounds or skin disease; and those who inject drugs or are experiencing homelessness.
